# Recommendations on how to achieve tobacco-free nations in Europe

**DOI:** 10.18332/tpc/110587

**Published:** 2019-07-18

**Authors:** Marc C. Willemsen, Bethany Hipple Walters, Daniel Kotz, Linda Bauld

**Affiliations:** 1 Department of Health Promotion, Maastricht University, Maastricht, Netherlands; 2 Netherlands Expertise Center for Tobacco Control, Trimbos Institute, Utrecht, Netherlands; 3 Addiction Research and Clinical Epidemiology Unit, Institute of General Practice, Heinrich-Heine-University Düsseldorf, Düsseldorf, Germany; 4 College of Medicine and Veterinary Medicine, Usher Institute, University of Edinburgh, Edinburgh, United Kingdom

**Keywords:** tobacco control, Europe, WHO FCTC, knowledge transfer, research capacity, capacity building

## Abstract

European countries vary widely in the development and implementation of effective tobacco-control programs and policies. Why some countries lag behind others is inherently a political matter. National-level policymakers struggle between the need to protect public health and the need to recognize economic and ideological considerations. Within this context, use of scientific evidence plays an important role in the policy making process. Articles 20 and 22 of the World Health Organisation’s Framework Convention of Tobacco Control (FCTC) oblige countries to develop and coordinate research on aspects of tobacco control and require of them to facilitate knowledge transfer and capacity building between countries. This paper considers various ways how EU and national policy makers may accomplish this. We conclude that progress in three areas is needed: 1) generation of more scientific evidence relevant for each country; 2) facilitation of policy learning between countries; and 3) building capacity and collaborations between researchers and tobacco-control advocates to bridge the gap from research to policy, especially in countries with weak tobacco-control infrastructures.

## INTRODUCTION

Tobacco use and exposure to tobacco smoke remain the leading causes of preventable death and disease in Europe. However, despite the strong need for public health interventions, countries in Europe vary widely in the development, implementation and research on effective tobacco-control programs and policies^1^. The need for tobacco-control programs and policies can be seen in high income countries, as well as in low- and middle-income countries (LMICs)^2^. A recent study concluded that if smoking were to be eliminated in the Nordic countries, a total of 430000 cancer cases could be avoided over a 30-year period^3^. Many policymakers and public health professionals in Europe see the need for effective, efficient and evidence-based tobacco-control measures. In 2017, the European Network for Smoking and Tobacco Prevention (ENSP) called for an end to the tobacco epidemic in Europe, which was operationalized as a reduction in the prevalence of current tobacco smoking to below 5% prevalence, defined as being smoke-free, by 2040^4^. The Finnish and Dutch governments have also stated the intention to become smoke-free by 2040^5,6^. The Irish government is more ambitious and aims to be tobacco-free by 2025^7^, while Scotland is aiming for 2034^8^. The French health minister is striving for ‘the first generation of non-smoking adults by 2032^9^.

The push towards tobacco-free nations is best achieved through the implementation of effective tobacco control. A comprehensive set of programs and activities are needed that include: 1) eliminating exposure to tobacco smoke, 2) increasing smoking cessation services, 3) implementing mass media campaigns, and 4) efforts to make tobacco use less appealing for consumers, which may include price increases (Table 1). European countries with more comprehensive tobacco policies generally have a greater reduction in smoking rates and higher quit ratios^10-12^. These four methods are the cornerstone of evidence-based and effective tobacco control and have been endorsed by the World Health Organisation (WHO) Framework Convention for Tobacco Control (FCTC), a global health treaty that represents the gold standard for tobacco-control policy^13^. Full implementation of the FCTC alone will not be sufficient to eliminate tobacco use and exposure^14^. However, in most countries there is still much room for improving the level of implementation of the FCTC package of interventions. This has the potential to significantly reduce tobacco use and exposure in the medium-to long-term while creating a social climate for the types of endgame strategies that are ultimately needed to achieve tobacco-free societies^15^.

**Table 1:** The cornerstones of a comprehensive approach to tobacco control

Eliminate exposure to tobacco products via: smoke-free environments point-of-sale display bans reduction of number of tobacco sale outlets Provide comprehensive and affordable nation-wide smoking cessation support systems for those who want to quit smoking Implement continuous media campaigns to raise awareness of the harms from smoking and to promote quit attempts Make tobacco products less attractive through: regular and substantial increases of the consumer price of tobacco through tax increases advertising and promotion restrictions health warnings on cigarette packs and plain packaging regulating additives in tobacco products that contribute to attractiveness and addictiveness

In 2005, the European Union (EU) and EU Member States ratified the FCTC treaty^16^. Although the treaty is legally binding by international law, there is no obligation to transpose the Articles from the treaty into national laws and there are no sanctions for non-compliance. It is thus left to the goodwill of governments, and ultimately, to non-governmental organisations (NGOs) to challenge governments through legal action for noncompliance with the articles of the framework. Most articles from the treaty leave room for interpretation, despite the existence of detailed guidelines to the main articles^17^. As a result, there are notable differences in the level of implementation of the various FCTC tobacco-control ‘building blocks’ among European countries^1^. The UK and Ireland perform best by having the highest cigarette prices, developing a smoking cessation treatment infrastructure, establishing comprehensive public place smoking bans, and having in place advertising bans and health warnings^1^. Austria, Germany, Luxembourg, and Greece, for example, have implemented significantly fewer recommendations. The reason why countries lag behind others is inherently political. EU member state policymakers struggle between the need to protect public health and the need to recognize economic and ideological considerations^18^.

Others have discussed strategies to strengthen implementation of FCTC through better research collaboration, but have not discussed this within a European context^19^. The aim of our paper is to analyse how EU and European national policy makers may accelerate the process of controlling tobacco use and exposure. Our discussion focuses on generating more country-specific scientific evidence, facilitating policy learning, and promoting capacity building in tobacco-control research, policy making, and program implementation.

### Generating country-specific scientific evidence

Policymakers are, in principle, dependent on the outcomes of research programs to inform strong national tobacco-control policies. While there is an extensive evidence-based body of knowledge on tobacco-control programs and strategies, further research is needed as tobacco use and exposure patterns change, as new programs are implemented, and as new products are developed (such as electronic cigarettes and heated tobacco products). Research is particularly important for gaining insight into the effects of policies and interventions, including monitoring national trends in the prevalence of tobacco use and mapping the attitudes of citizens towards tobacco and tobacco-control policies. The availability of scientific evidence has been acknowledged by international experts as having had a ‘substantial’ impact on the adoption of clean indoor air policy, taxation and cessation treatment policy, and a ‘modest’ effect on other policy areas^20^. The need for well-developed science-informed arguments is more urgent than ever, since the tobacco industry repeatedly questions tobacco policies for lacking an evidence base, as illustrated by recent attempts to discredit standardised packaging in the UK^21,22^.

In order to be most effective, country-specific studies can be particularly useful in making the case for policy change. The EU consists of 28 countries, with diverse cultural and political orientations and economic conditions. This diversity results in unique policy environments for tobacco control^23^. For example, the relatively late implementation of smoke-free public places in the Netherlands has been explained by the attitude, among policymakers and the public, to be considerate to smokers, which reflects national cultural values of individualism and egalitarianism^18^. Tobacco-control policy making in the Netherlands is influenced by a strong tradition of corporatism where it is customary to involve societal organisations, including industry representatives, in the policy making process, which tends to slow down the adoption and implementation of new tobacco-control measures. Country-specific research can meet the needs of each country’s unique political, cultural and healthcare system settings.

Article 20 of the FCTC obliges the EU and national governments to develop and coordinate research on aspects of tobacco control (Table 2). In addition, Article 22 requires countries to facilitate knowledge transfer and capacity building within the network of the FCTC Parties. Recently, European health organisations and tobacco-control researchers have called on the EU and its Member States to better implement FCTC Articles 20 and 22 and improve cross-European research, surveillance, and knowledge exchange on tobacco control^24^. This group advised the EU to set up a European Centre of Excellence on Tobacco Control, that could help develop and coordinate the research needed to accelerate tobacco control across the EU. This follows a similar call from 2004^25^, but such a centre of excellence has not yet materialised. A pan-European centre could build upon effective consortia in individual countries, such as the 13-university UK Centres for Tobacco and Alcohol Studies^26^ and the Netherlands Network for Tobacco Research (NNvT)^27^.

**Table 2:** Key elements in the FCTC Article 20 on research and surveillance

Parties to the treaty (i.e. governments) shall develop and promote national research in the field of tobacco control. Parties shall initiate and cooperate in the conduct of research and scientific assessments, and in so doing promote and encourage research that addresses the determinants and consequences of tobacco consumption and exposure to tobacco smoke. Parties shall promote and strengthen training and support for all those engaged in tobacco-control activities, including research, implementation and evaluation. Parties shall establish programmes for national, regional and global surveillance of the magnitude, patterns, determinants and consequences of tobacco consumption and exposure to tobacco smoke.

Research funding within Europe is fragmented^25^ and research output is unevenly distributed, with Eastern European countries being particularly depleted of scientific underpinning of tobacco control^28^. Some of the research outputs from these countries do not get into international databanks such as PubMed and Scopus. This can occur when the research is published in non-indexed national journals or in journals that are aimed at clinicians rather than researchers. Furthermore, the uneven distribution of research across Europe is a result of the limited capacity for writing funding proposals and limited national sources for research funding, especially in Eastern European countries.

International grants reward those with an established record of successful grants in the past, access and familiarity with the most recent literature in tobacco control, and, arguably, a very good command of English^29^. Many researchers prefer to work with existing partners and institutions, which may make it difficult for new tobacco-control researchers to participate in research consortia. Furthermore, the type of research that is needed most to advance FCTC has not received sufficient funding, evidenced by relatively few publications on population-level tobacco-control policies^28^. This is concerning since policy makers working in government departments relevant to tobacco control need data on effective measures in tobacco control, such as what tobacco taxation increases are feasible and under which conditions will these have an optimum impact on smoking rates. Research funding is all the more important now, in light of recent tactical manoeuvres by the tobacco manufacturer Philip Morris to have poorly-resourced research institutes accept research money from their well-funded Foundation for a Smoke-free World^30^.

While some funding from the EU Research and Innovation funding programmes (FP7 and Horizon 2020) has been spent on studies relevant to tobacco-control research, there is currently no dedicated fund to support an infrastructure for tobacco-control research for the EU, as distinct from individual projects. This makes it extremely difficult for researchers to find EU subsidies for research that can support tobacco control at the European level. More strategic funding for tobacco-control research is therefore needed. The EU new FP9 Horizon Europe proposal for 2021–2027 includes support for research infrastructures, which could be helpful. In addition, few individual countries in Europe have formally coordinated tobacco research strategies or dedicated research budgets, indicating that the EU has an important and distinct role to play in this area.

Finally, the EU could play an important role in supporting tobacco-control research networks that can bring together young and established researchers from across Europe with a focus on supporting LMICs in Europe. To do this most effectively, it could build on existing scientific networks. In order to narrow the evidence–policy gap, the following actions may be taken:

- Where possible, research should be country-specific, while meeting international research standards and being eligible for publication in open-access journals.
- Research that is needed most to advance FCTC, such as on population-level interventions, is not receiving sufficient funding. Increased funding is therefore needed.
- Dedicated budgets for tobacco-control research are needed, both at the national and EU level.
- Funding within Europe is fragmented, with few research outputs emerging from LMICs. Specific funding is needed to develop and extend national research infrastructures.
- EU support for the creation of a European Centre of Excellence on Tobacco Control.
- EU support for tobacco-control research networks and research training programs.

### Facilitating policy learning

Processes of policy convergence are accelerated by policy learning and policy transfer^31^. In some countries, failure to advance can be attributed to insufficient knowledge about what works best, but in most countries it is predominantly a political matter^32^. The need for tobacco control can be appreciated in different ways, emphasising different aspects of the problem and its various solutions, such as health impact, effectiveness, moral aspects, ideological considerations, costs, and economic consequences. The complexity of tobacco control sometimes leads to confusion and ambivalence among policy makers about what to do. The message for stronger tobacco control to national policy makers may have more impact if tobacco-control strategies were framed in a clear, unambiguous manner to reduce confusion and to help policymakers and politicians feel more comfortable with tobacco-control solutions. For example, in an increasing number of countries, the idea of protecting children from the harms of tobacco (‘a smoke-free generation’) serves as a catalyst for tobacco control that has both societal and political support. It is precisely in this area of finding the best advocacy strategy and how to build effective national advocacy coalitions that national tobacco advocacy organisations and health ministries in countries with less advanced tobacco-control regimes would benefit from international support and bestpractices examples. Existing networks have created some helpful opportunities for diffusion and transfer of knowledge; these networks also explicitly try to include researchers from LMICs. Having advocacy and policy tracks at scientific conferences is a way to get policy makers more interested in research findings and making researchers more sensitive to policy needs. These awards can help built CVs of early career researchers.

We recommend the following steps to facilitate information exchange between countries and between policymakers and researchers:

- The EU should stimulate policy learning across countries by supporting EU-wide policy learning platforms such as conferences, expert meetings and webinars, with a special focus on how to translate research findings for policy makers.
- Tobacco-control researchers should work to disseminate their findings in clear language to a large audience through infographics, information sheets, videos, and informational interviews.
- Established tobacco-control researchers should mentor new researchers, especially researchers from LMICs, in the development of research notes, press releases, and summaries for policy makers.
- The EU to support policy learning in tobacco control by disseminating clear, easy-to-use recommendations for how to use findings from tobacco control in policymaking.
- The EU should support and moderate meetings between tobacco-control researchers and policymakers; these meetings would serve as information sessions and would facilitate lasting collaborations.

### Supporting capacity building

Since the key to accelerating the process of eliminating the tobacco epidemic is largely in the hands of policy makers, national tobacco-control advocacy coalitions need to work together to develop a strong and comprehensive approach to tobacco control with a long-term aim. The process of reducing tobacco use and exposure would thus benefit from increased collaboration between researchers and national tobacco-control advocacy coalitions. Researchers often do not have the skills to directly use their research to influence policy and, often, active lobbying by researchers is deemed inappropriate. In contrast, tobacco-control advocacy organisations do have these skills and are able to broker meetings or establish relationships with policymakers. However, advocates may not be well-versed in interpreting study methods or findings. Stronger collaborations between researchers and advocates could help bridge these gaps and ensure that research is communicated effectively.

Implementation of the FCTC measures depends on a country’s capacity to implement, with LMICs often lacking essential elements^33^. These include a strong organisational infrastructure that includes different types of organizations with actors who have financial resources, close connections to researchers and access to data and evidence from research^34^. Other important factors are having long-term collaboration and trust, frequent information exchange, and strong leaders who understand the policy process. Such factors need to come together to produce synergy, making collaborations stronger and more effective^35^. This synergy could be supported by the European Commission; the EC announced programs to support capacity building for tobacco control in the EU countries^36^, but there are still no large scale programs or funding. The EU may look at successful initiatives from outside the EU. The UK government has supported the Tobacco Control Capacity Programme, led by academics but involving NGOs and policy makers in the UK, Africa and South Asia through the Global Challenges Research Fund^37^.

In summary:

- Stronger collaborations between tobacco-control researchers and advocacy organisations within countries and at the EU level are required and could build capacity in both types of organisations to enhance research communication, particularly to policy-makers.
- The EU should, in turn, stimulate collaboration between tobacco-control advocacy organisations and national-level policy makers
- The EU is advised to initiate programs to strengthen tobacco-control advocacy capacity in LMICs, for example through supporting access to journals (open-access policies), support meetings and conferences in LMICs, create partnerships between established and young researchers in LMICs, and finance online courses and information sessions.

## DISCUSSION

Disputes over tobacco control are fought within changing policy environments and differ substantially between countries in Europe. As a result, large dissimilarities exist in level of implementation of the FCTC measures. Business-as-usual, which in many countries comes down to small periodic tax hikes, some level of health information provision, and some provision of smoking cessation support, will simply not be enough^38,39^. To accelerate the implementation of more effective tobacco control, progress in three areas is needed: generation of more scientific evidence relevant for each country, facilitation of policy learning among countries, and capacity building and building collaborations between researchers and tobacco-control advocates in countries that currently have weak tobacco-control infrastructures.

The European Commission has a substantial impact on an individual member state’s tobacco policy through issuing Directives, which are legally binding and must be transposed into national law. However, the EU can do more by actively coordinating tobacco-control research at EU level, reducing research inequalities between countries. To accomplish this, tobacco-control research networks and capacity building training programs should be improved and partly developed, so that researchers are trained in research communication and working with advocacy colleagues, and advocacy colleagues are offered training in understanding, interpreting and communicating research findings to policy-makers. Tobacco research should be given a dedicated budget line in the new €100 billion research and innovation Framework Programme (FP9), Horizon Europe, the successor to Horizon 2020.

The topic of how to support countries with accelerating implementation of FCTC is a timely issue. When governments recently met at the 8th session of the Conference of the Parties (COP) to the Framework Convention on Tobacco Control (FCTC) in Geneva, they agreed on the urgent need for strategic planning to ensure accelerated action on tobacco control. A Medium-Term Strategic Framework (MTSF), also known as the Global Strategy to Accelerate Tobacco Control, was adopted. This aims to support countries with adapting tobacco-control activities to meet local needs, among others through building partnerships with civil society and other actors.

## CONCLUSIONS

Our paper gives concrete recommendations on how to achieve tobacco-free nations in Europe and most of these are also applicable globally.
